# Differential toxicities of fine particulate matters from various sources

**DOI:** 10.1038/s41598-018-35398-0

**Published:** 2018-11-19

**Authors:** Minhan Park, Hung Soo Joo, Kwangyul Lee, Myoseon Jang, Sang Don Kim, Injeong Kim, Lucille Joanna S. Borlaza, Heungbin Lim, Hanjae Shin, Kyu Hyuck Chung, Yoon-Hyeong Choi, Sun Gu Park, Min-Suk Bae, Jiyi Lee, Hangyul Song, Kihong Park

**Affiliations:** 1 School of Earth Sciences and Environmental Engineering, Gwangju Institute of Science and Technology (GIST), Gwangju, Republic of Korea; 2 Department of Environmental Engineering, Anyang University, Anyang, Republic of Korea; 3 Department of Environmental and Global Health, University of Florida, Gainesville, FL USA; 4 Department of Industrial Plant Science & Technology, Chungbuk National University, Cheongju, Republic of Korea; 5 R&D Headquarter, KT&G, Daejeon, Republic of Korea; 6 School of Pharmacy, Sungkyunkwan University, Suwon, Republic of Korea; 7 Department of Preventive Medicine, Gachon University Graduate School of Medicine, Incheon, Republic of Korea; 8 Department of Environmental Engineering, Mokpo National University, Muan, Republic of Korea; 9 Department of Environmental Science and Engineering, Ewha Womans University, Seoul, Republic of Korea

## Abstract

Fine particulate matters less than 2.5 µm (PM_2.5_) in the ambient atmosphere are strongly associated with adverse health effects. However, it is unlikely that all fine particles are equally toxic in view of their different sizes and chemical components. Toxicity of fine particles produced from various combustion sources (diesel engine, gasoline engine, biomass burning (rice straw and pine stem burning), and coal combustion) and non-combustion sources (road dust including sea spray aerosols, ammonium sulfate, ammonium nitrate, and secondary organic aerosols (SOA)), which are known major sources of PM_2.5_, was determined. Multiple biological and chemical endpoints were integrated for various source-specific aerosols to derive toxicity scores for particles originating from different sources. The highest toxicity score was obtained for diesel engine exhaust particles, followed by gasoline engine exhaust particles, biomass burning particles, coal combustion particles, and road dust, suggesting that traffic plays the most critical role in enhancing the toxic effects of fine particles. The toxicity ranking of fine particles produced from various sources can be used to better understand the adverse health effects caused by different fine particle types in the ambient atmosphere, and to provide practical management of fine particles beyond what can be achieved only using PM mass which is the current regulation standard.

## Introduction

Fine particles in the ambient atmosphere are of significant research interest owing to their impacts on climate change and detrimental effects on human health. These particles are directly emitted from various sources and produced by gas-to-particle conversion of secondary products from atmospheric oxidation of SO_2_, NO_x_, and hydrocarbons. Epidemiological and toxicological studies worldwide have suggested a strong link between exposure to fine particles and adverse health effects (e.g., respiratory disease, lung cancer, cardiovascular disease, and premature mortality)^1–3^. The World Health Organization (WHO) established guidelines for particulate matter based on mass concentration of particulate matter less than 2.5 µm (PM_2.5_)^4^. However, health risks of PM_2.5_ are not fully taken into account with the PM_2.5_ mass, and all fine particles may not be equally toxic^5^. Due to multiple sources and formation pathways, ambient PM_2.5_ have diverse sizes, shapes, surface charges, surface chemistry and chemical compositions, leading to differential health effects among particle types. Even different PM_2.5_ with the same mass concentrations may exert variable effects on human health. For example, an earlier study by Lelieveld *et al*.^3^ in their assessment of the contribution of outdoor air pollution sources to premature mortality reported that health impact was dependent on assumptions on toxicity of particles^6,7^. A recent review on epidemiological and toxicological literatures related to long-term effects of PM components or source factors suggests that there has been insufficient information to make clear conclusions about differential health effects among components or sources^8^. It was also reported that much more enhanced understanding of exposure and health effects should be needed before it can be concluded that the control of specific sources or components of PM_2.5_ should be more effective to protect human health than the PM_2.5_ mass as a whole^9^.

The differential toxicities among particle types according to chemical composition remain to be established due to the existence of numerous chemical constituents in ambient aerosols. Conduction of complete toxicity tests for all possible chemical constituents of ambient aerosols and generation of practical chemical groups for regulatory purposes is a virtually impossible task. Moreover, it is difficult to ascertain the combined roles of individual chemical constituents (e.g., synergetic effects between chemical species and transformation of chemicals through complex cellular mechanisms) in triggering adverse health effects. Thus, determination of source-specific toxicity may present a more efficient alternative means to elucidate the effects of individual PM_2.5_ rather than chemical component-specific toxicity measurements. The number of toxicity tests for hundreds or thousands of chemical components in PM_2.5_ (chemical component-specific toxicity test) can be reduced to the smaller particle groups that are major distinct sources for fine particles (source-specific toxicity test) and a range of biological responses to source-specific aerosols under comparable conditions (PM generation, collection, exposure and biological systems) can be assessed. Typically, source apportionment studies are effectively used to determine the major sources for ambient PM_2.5_ through measurement of their chemical components^10,11^. Source apportionment results can be combined with source-specific toxicity data to evaluate overall toxicity of ambient PM_2.5_.

**Figure 1:**
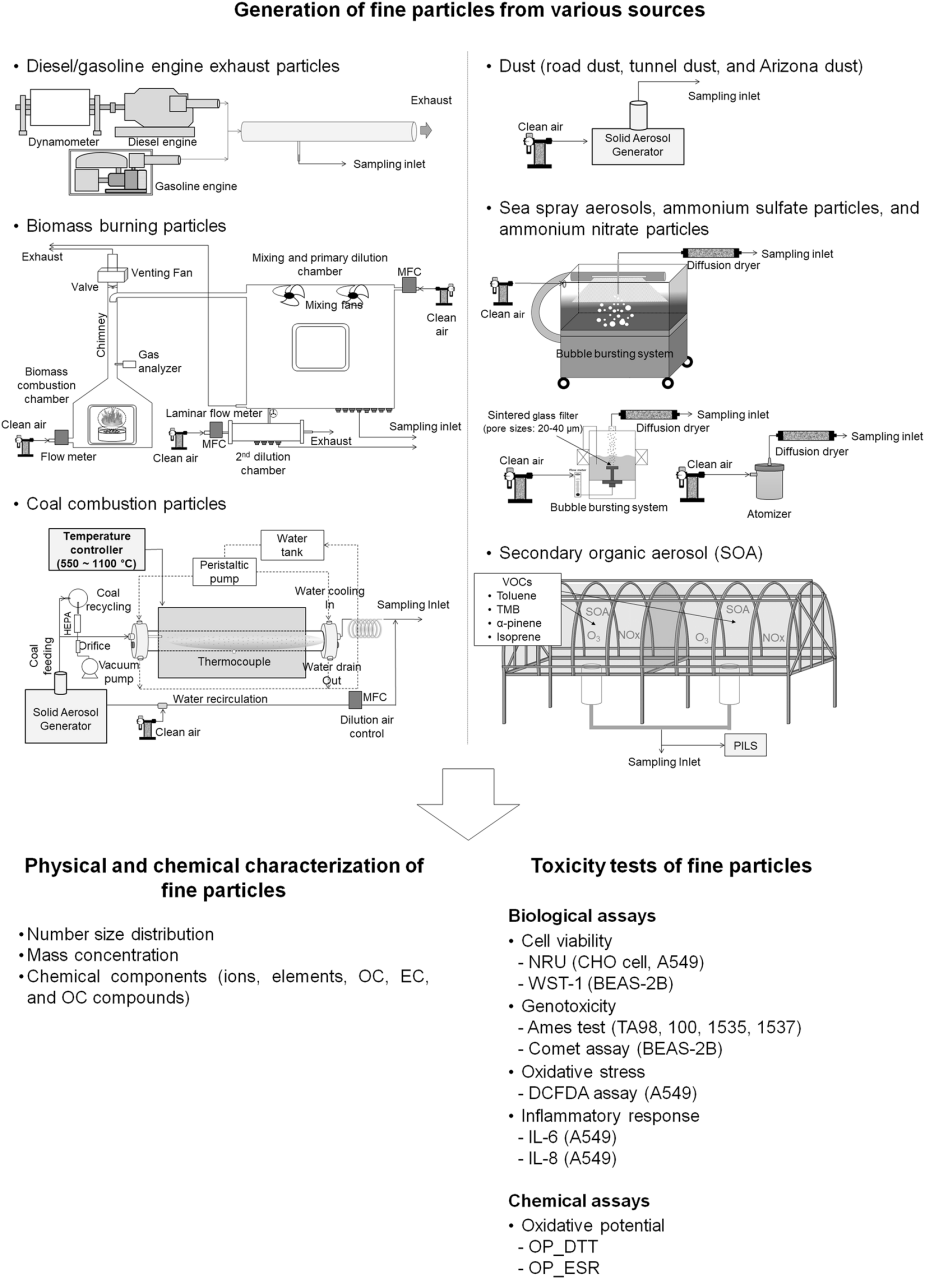
Experimental design for generation, physical and chemical characterization, and toxicity tests for various primary and secondary aerosols from different sources.

The purpose of this study is to assess variability in toxicities of fine particles produced from various combustion sources and non-combustion sources which are known major sources of PM_2.5_ (Fig. 1). The combustion sources include diesel engine, gasoline engine, biomass burning and coal combustion. The non-combustion sources include road dust, sea spray aerosols, ammonium sulfate, ammonium nitrate, and secondary organic aerosols (SOA) produced from the photo-oxidation of toluene, 1,3,5-trimethylbenzene (TMB), isoprene, and α-pinene in the presence of NO_x_ under natural sunlight. To determine toxicity of various particles, multiple biological and chemical endpoints (oxidative potential (OP), cell viability, genotoxicity (based on mutagenicity and DNA damage), oxidative stress and inflammatory response) using human airway cell lines, animal ovary cell lines and *Salmonella* strains with preexisting mutations were determined. This method facilitated the direct comparison of various endpoints linked to health effect on respiratory system with minimization of differences in exposure and biological systems. It is believed that the developed toxicity score accounts for differential toxicities of various aerosols linked to adverse health effects on respiratory system. The tested method assigned priority to PM_2.5_ sources potentially harmful to human respiratory system. However, the dominant mortality also came from cardiovascular diseases^12^. Further incorporation of toxicological data which are related to health effects on cardiovascular system could improve the toxicity score database. The multiple endpoints were integrated to derive toxicity scores for particles originating from different sources. The database for toxicity could be used to better understand health effects caused by different fine particle types of ambient PM_2.5_. Chemical characterization of the particles was additionally conducted to relate their major chemical components to the toxicity score for source-specific aerosols.

## Methods

### Generation of fine particles from various sources

Exhaust particles were produced from a heavy duty diesel engine (DL08S, 7640 cc, 1500 rpm, Doosan Infracore, Korea), light duty diesel engine (DL08S, 2740 cc, 1500 rpm, Isuzu motors, Japan), diesel generator engine (192FC, 498 cc, 3000 rpm, Hi-earns, China) and gasoline generator engine (GXH50, 50 cc, 5500 rpm, Honda, Japan). Table S7 summarizes the specifications of the diesel and gasoline engines used in this study. The engine exhaust particles were diluted (1:100) by an aerosol diluter (3302A, TSI Inc., USA) and sampled into PM_2.5_ filters for determination of chemical composition and toxicity, and real-time aerosol instruments were used to assess the number size distribution and mass concentration, as shown in Fig. 1.

A biomass burning chamber mainly consisting of a combustion chamber (0.54 m^3^), primary dilution chamber (3.75 m^3^) and secondary dilution chamber (0.04 m^3^), was employed for generation of rice straw and pine stem burning particles (Fig. 1). Clean air was supplied into the combustion chamber using a mass flow controller. About 25 g of biomass was loaded on the grid of the combustion stove and ignited using a propane torch. Rice straw and pine stems for biomass were collected from rural and forest areas in Korea. The smoke was drawn into a primary (3.75 m^3^) dilution chamber (1:1), followed by a secondary dilution chamber (0.04 m^3^) (1:10). Several outlets were used for PM_2.5_ sampling on filters for chemical and toxicity analyses and for real-time measurements of number size distribution and mass concentration with further dilution (1:100).

A bench scale high-temperature furnace (HTF55342C, Thermo Electron Corp., USA) with a quartz tube was employed to produce coal combustion particles (Fig. 1). Bituminous coal used by coal power plants (Korea South Power Co. Ltd., Hadong, Korea) was pulverized and sieved through a 200-mesh screen (< 75 µm). Pulverized bituminous coal was fed into a quartz tube (0.00327 m^3^) using a solid aerosol generator (SAG 410, Topas GmbH, Germany) at a feeding rate of 3.5 g/h. Several burning temperature conditions (550, 900, and 1100 °C) were used to generate coal combustion particles. Next, particles from the furnace were diluted with clean air and introduced into filters for determination of chemical composition and toxicity, and real-time aerosol instruments were used for determination of number size distribution and mass concentration. Prior to real-time measurements, samples were further diluted (1:100) using an aerosol diluter.

Road dust was collected from the shoulder of the roadside and walkway in a tunnel near a junction area in Korea (urban Gwangju). All collected samples were dried in a desiccator for 24 h. A 12-mesh sieve (< 1.7 mm) was used to remove large particles, and the dust further sieved through a 400-mesh screen (< 38 µm) to collect fine dust samples. Sieved dust was aerosolized using a solid aerosol generator under identical conditions with an input pressure of 15 psi and feeding rate of 6 g/h, as shown in Fig. 1. Aerosolized dust was subsequently introduced into the PM_2.5_ filter sampling system (Fig. 1). Prior to real-time measurements, aerosols were diluted (1:100) using an aerosol diluter. Carbon black powder (Cabot Corp., USA), Arizona dust^13^ and Mongolian dust collected from roadsides in urban Ulaanbaatar were additionally re-suspended using the above method.

Sea spray aerosols were generated from natural seawater sampled from the South sea of Korea using two system types, specifically, a marine aerosol reference tank (MART) (Marine Research Systems, USA) and a prototype bubble bursting chamber (Fig. 1). Ammonium sulfate (Sigma-Aldrich, USA) and ammonium nitrate (Sigma-Aldrich, USA) in deionized water solution were used to produce ammonium sulfate and ammonium nitrate particles with the aid of an atomizer, as shown in Fig. 1. Particles were dried using a series of diffusion driers before sampling into filters or introducing into real-time aerosol instruments.

Secondary organic aerosols (SOA) were produced under natural sunlight using University of Florida Atmospheric PHotochemical Outdoor Reactor (UF-APHOR) dual chambers (52 m^3^ for each) located on the roof of the Black Hall at the University of Florida (Fig. 1). Before sunrise, hydrocarbon was inserted into the chamber with a U-shaped injector. For photooxidation of toluene, HONO produced from the reaction of 0.1 M NaNO2 and 10% w/w H2SO4 aqueous solution were introduced into the chamber as a source of OH radicals. The initial mixing ratio of HONO was estimated based on the difference in NO_2_ concentrations with and without the base denuder (1% Na_2_CO_3_ + 1% glucose)^14^. Particle size distribution and concentrations of chamber-generated SOA were monitored with the aid of a scanning mobility particle sizer (SMPS) and calculated to mass concentrations using SOA density (1.3 g/cm^3^ for α-pinene SOA and 1.4 g/cm^3^ for other three types of SOA)^15–17^. The mass concentration of organic carbon (OC) was additionally monitored using a semi-continuous Organic carbon/Element carbon (OC/EC) aerosol analyzer. Organic matter (OM) concentration was determined by multiplying OC by the [OM]/[OC] ratio, which was calculated as 1.6 for α-pinene and 2.0 for the other three SOA types^18^. The chamber experiment conditions used to produce various organic aerosols and SOA yields are summarized in Table S2. Generated SOAs were collected using a Particle-Into-Liquid sampler (PILS) (Applikon, Netherlands) and toxicities were examined via chemical and biological assays.

### Physical and chemical characterization of fine particles

Number size distribution of fine particles from various sources was measured with a scanning mobility particle sizer (SMPS) (3081 DMA and 3022A CPC, TSI Inc., USA) (14 to 660 nm), NanoScan SMPS (3910, TSI Inc., USA) (10 to 420 nm), and optical particle sizer (OPS) (3330, TSI Inc., USA) (0.3 to 10 μm). PM_2.5_ mass concentration was assessed with a Dust Trak instrument (DRX, TSI Inc., USA). For offline chemical analysis, PM_2.5_ filter samples (Zefluor filter for ions and elements, and quartz filter for carbonaceous species) were collected from various sources in laboratory and urban (Gwangju), rural (Jangseoung), roadside (Gwangju), and industrial (Gwangyang) sites in Korea.

Ions, elements, and carbonaceous species (OC and EC) in PM_2.5_ filter samples were determined via ion chromatography (IC) (850 Professional IC, Metrohm, Switzerland), inductively coupled plasma-mass spectrometry (ICP-MS) (7500ce, Agilent, USA), and an OC-EC carbon analyzer (5L, Sunset laboratory Inc., USA), respectively. A Zefluor filter was used for ion and element analyses. The filter was equilibrated at a temperature of 21 ± 2 °C and relative humidity of 35 ± 5% for 24 h before and after sampling. Triplicate pre- and post-weighing was performed for each filter. After sampling, the Zefluor filter was cut into two equal parts for analyses of ions and elements. One part of the filter was extracted with 20 mL deionized water under 1 h ultra-sonication and 3 h shaking. The water extracts were subsequently passed through a polytetrafluoroethylene (PTFE) syringe filter, and 8 water-soluble ions (SO_4_^2−^, NO_3_^−^, Cl^−^, NH_4_^+^, Na^+^, K^+^, Mg^2+^, and Ca^2+^) analyzed via IC. Recovery was determined as 87.5–103.5% using standard samples. The second portion of the sampled filter was digested in PTFE (polytetrafluoroethylene) vessels by adding HNO_3_ and HCl (3:1) in a closed microwave system. The digested solution was concentrated to 0.5 mL in a heating block and treated with 2% HNO_3_ up to a volume of 10 mL. The solution was subsequently filtered using a PTFE syringe filter and 14 elements (Al, As, Ba, Cd, Co, Cu, Fe, Mn, Ni, Pb, Sr, Ti, V, and Zn) were analyzed via ICP-MS. Recovery was determined as 87.8–118.8% using standard samples. A quartz filter (Pall corporation., USA) was applied for analysis of carbonaceous species. The filter was prebaked at 450 °C for 5 h before sampling and stored in a freezer at −20 °C prior to and after sampling. OC and EC were measured with the OC-EC analyzer employing the thermal-optical transmittance (TOT) method^19^ based on the National Institute for Occupational Safety and Health (NIOSH) 5040 temperature protocol^20^.

For analysis of organic compounds for biomass burning particles, PM_2.5_ samples were extracted with dichloromethane (DCM) followed by acetone using a Soxhlet extractor. The solvent extract was subsequently reduced to a volume of 250 μL with a rotary evaporator and nitrogen blowdown. Then, the extract was methylated using diazomethane (1-methyl-3-nitro-1-nitrosoguanidine, MNNG). It was reacted with silylation reagent to derivatize levoglucosan to trimethylsilyl derivatives (TMS-derivatives). Eight classes of organic compounds (n-alkanes, hopanes and steranes, PAHs, n-alkanoic acids, benzoic acid, dicarboxylic acids, resin acid and levoglucosan) and 122 organic species were analyzed using GC-MS (7890A GC, 5975C MSD, Agilent Technologies, USA). For organic analyses of coal combustion, diesel and gasoline engine exhaust particles, PM_2.5_ samples were extracted using the mixture of DCM and methanol (MeOH) (3:1 by volume) with 1 h sonication. The extract was reduced to a volume of 500 μL with a Turbo Vap concentrator (Turbo Vap II, Caliper Life Sciences, USA) subjected to a gentle stream of nitrogen (99.9%). Six classes of organic compounds (n-alkanes, hopanes, PAHs, n-alkanoic acids, dicarboxylic acids, and levoglucosan (61 organic species)) for coal combustion particles and four classes of organic compounds (n-alkanes, PAHs, n-alkanoic acids, and levoglucosan (49 organic species)) for diesel and gasoline engine exhaust particles were analyzed via GC-MS (7890A GC, 5975C MSD, Agilent Technologies, USA).

### Biological and chemical responses of fine particles

For assessment of particle toxicity, various chemical and biological assays were conducted. PM_2.5_ samples on glass fiber filters and PTFE filters (Pall corporation, USA) were extracted using DI water and dimethylsulfoxide (DMSO) (Sigma Aldrich, USA) at room temperature and dichloromethane (DCM) (Samchun chemicals, Korea) at 37 °C. For the chemical assay, OP (used to estimate the capability of PM to generate reactive oxygen species (ROS)) was measured. ROS are reported to cause oxidative stress followed by inflammation and cell death^21,22^. The OP was measured using two different techniques: Dithiothreitol (DTT) chemical assay (OP_DTT)^23,24^ and Electron Spin Resonance spectrometry (OP_ESR)^25,26^. OP_DTT evaluates the reaction of ROS via the formation of a DTT-disulfide as a result of electron transfer from DTT to ROS. DTT is commonly used as a chemical surrogate for cellular reducing agents (NADH and NADPH) in order to mimic *in vivo* interactions of PM with biological oxidants. On the other hand, OP_ESR measures the ability of PM to generate OH radicals via the Fenton oxidation in the presence of H_2_O_2_ (hydrogen peroxide) and 5,5-dimethyl-1-pyrroline-N-oxide (DMPO) as a spin trap agent. OP activities measured using both assays were employed to evaluate the ROS generation capability of PM samples.

For the biological assay, human airway epithelial (A549, H292, BEAS-2B and SAEC) and Chinese hamster ovary (CHO-K1) cell lines were used to assess *in vitro* cell toxicity. A variety of biological responses (cell viability, genotoxicity (mutagenicity and DNA damage), oxidative stress, and inflammation) that facilitate identification of the specific PM triggering ROS and inflammatory responses leading to oxidative stress, genotoxicity and cell death were evaluated for source-specific aerosols^27–29^. Neutral red uptake (NRU) (Sigma Aldrich, USA)^30^ and water-soluble tetrazolium salt (WST-1) assays (Takara, Japan) were utilized to determine cell viability in the presence of PM_2.5_. Ames test^31^ and the Single Cell Gel Electrophoresis (SCGE) Comet assay^32^ were conducted to determine mutagenicity and DNA damage of cells induced by PM, respectively. Levels of cellular reactive oxygen species (ROS) causing oxidative stress in cells were measured via the DCFDA (2′,7′-dichlorofluorescein diacetate) assay (Abcam, UK)^33^. Measurements of IL-6 and IL-8 (Abcam, UK) were obtained for determining the expression of genes related to the inflammatory response^34^. The initial PM_2.5_ extracts were diluted 1–150 μg/mL for NRU, 10–500 μg/mL for WST-1, 0.1–1,000 μg/mL for the Ames test, 62.5–500 μg/mL for Comet, 15–150 μg/mL for DCFDA, and 2–15 μg/mL for the IL-6 and IL-8 assays while undiluted extracts were used for OP assays. Table S8 and Supplementary Information provides details of the chemical and biological assays performed in this study. The biological effects of PM are not comparable among different studies owing to distinct exposure concentrations, biological models, endpoints, and PM generation methods^35^. Here, we employed similar exposure and cell conditions and identical endpoints for various aerosols within the same batch to obtain comparable toxicity data for PM_2.5_ from different sources. However, non-linear concentration-response functions for various endpoints and different exposure concentrations will limit the ability to use toxicological data to predict risks in human populations. Experimental design for generation, physical and chemical characterization, and toxicity tests for various primary and secondary aerosols from different sources is illustrated in Fig. 1.

### Statistical information

Statistical analysis of all toxicity data performed using SPSS ver. 21.0 (IBM SPSS, USA). Differences between groups were assessed with Tukey’s post-hoc test following one-way analysis of variance (ANOVA). Statistical significance was accepted at *p* < 0.05.

## Results

**Figure 2:**
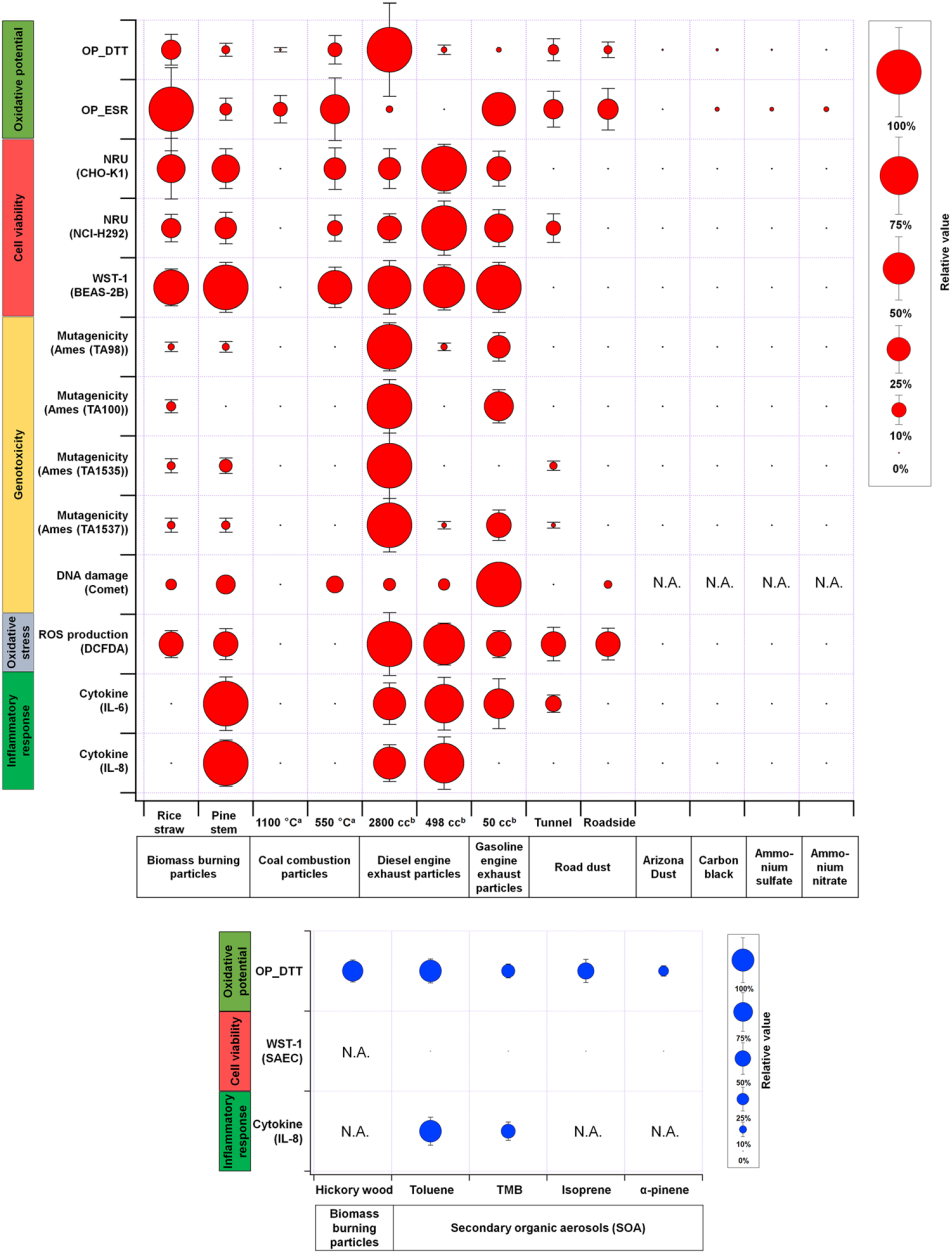
Multiple chemical and biological responses (oxidative potential, cell viability, genotoxicity (based on mutagenicity and DNA damage), oxidative stress and inflammatory response) for source-specific aerosols. The relative magnitude is plotted as the size of sphere with standard deviation, and the highest value among different aerosol types for each response is taken as 100% (maximum sphere size). ^a^Coal combustion temperature. ^b^Engine displacement.

For source-specific aerosols, Fig. 2 illustrates multiple chemical and biological responses (OP, cell viability, genotoxicity and DNA damage, oxidative stress, and inflammatory response). The toxicological values employed were OP activity normalized by PM_2.5_ mass for OP_DTT using dithiothreitol (DTT) assay and OP-ESR using electron spin resonance (ESR), half-maximal effective concentration (EC_50_) for cell viability, specific activity (number of revertant colonies per PM_2.5_ mass) for mutagenicity, significant concentration-dependent induction factor (summation of fold change relative to control divided by dose concentration within the concentration range showing a significant dose-response relationship) for DNA damage, relative fluorescence intensity (fold change relative to control for specific dose concentration) for oxidative stress, and relative maximum cytokine production (maximum fold change relative to control divided by dose concentration from the dose-response curve) for inflammatory response. The dose concentrations among sources were in a similar range for each assay except OP. For each endpoint, relative toxicological values of source-specific aerosols were plotted as sphere size with standard deviations. The highest value among the different aerosol types for each response is taken as 100% (maximum size of the sphere). All measurements of endpoints for source-specific aerosols, except SOAs, were conducted under similar conditions to facilitate direct comparison of the contributions of the different particles to biological and chemical responses.

Our results disclosed higher toxicity of combustion than non-combustion aerosols. Among the combustion aerosols, diesel engine exhaust particles (engine displacement of 2800 cc) were identified as the most toxic based on chemical and biological responses. Further analyses are required to confirm the statistical significance of this result as discussed in a toxicity score calculation section. In particular, genotoxicity (mutagenicity) and OP_DTT of diesel engine exhaust particles were significantly higher than those for other aerosol types. The mutagenic effects of soot particles are suggested to be associated with the organic components (e.g., PAH) generating reactive oxygen species (ROS) that are able to break DNA strands^36^. Polar or quinone fractions of PAH in diesel engine exhaust particles are reported to play an important role in the increased toxic response^37^. OP_DTT is sensitive to the amount of quinone, an oxidation product of PAHs and redox recycling agent in particles^25,38^. The mutagenicity of particles produced from smaller diesel engines (engine displacement of 498 cc) that emit lower levels of organic components was relatively low compared to particles from larger engines (2800 cc). A more in-depth explanation of chemical components is provided in a later section. Gasoline engine exhaust particles also showed comparable or lower toxicity relative to diesel engine exhaust particles based on various endpoints.

Both rice straw and pine stem burning particles (popular biomasses used in East Asia) showed significant toxicity in terms of effects on cell viability (NRU and WST-1) comparable to that of diesel engine exhaust particles. Pine stem burning particles were associated with higher inflammatory responses than rice straw burning particles. However, OP_ESR and OP_DTT were higher for rice straw particles. OP_DTT and OP_ESR is reported to be correlated well with OC and trace elements (Cu and Zn), respectively^25,26^. Rice straw particles contained higher fractions of Cu, Zn, and OC than pine stem particles, explaining the higher OP values obtained. Evaluation of the OP of PM_2.5_ using multiple assays is essential to provide useful complementary information.

In the case of coal combustion particles (burned at 1100 °C and 550 °C), the biological responses were relatively low compared to other combustion particle types. The bituminous coal was selected because it was the most abundant type used in the world^39^. Also, it has been extensively used in power plants in East Asia^40,41^. Variability in toxicity among different coal types was found to be small^42,43^. Two burning temperatures were used for simulation: residential coal combustion (low temperature) and power plant coal combustion (high temperature). Coal combustion particles generated at a temperature of 550 °C showed relatively higher toxicity in many endpoints than those at 1100 °C. One potential explanation for this finding is that the higher burning temperature leads to more complete burning of pulverized coal, leading to lower emission of carbonaceous species. Our data suggest that residential coal combustion particles typically emitted at lower burning temperatures are more toxic than power plant coal combustion particles emitted at higher burning temperatures. Levels of carbonaceous species, including toxic organic compounds (such as PAH), were significantly higher in residential than industrial coal combustion particles^44^.

Ammonium sulfate and ammonium nitrate particles which were aerosolized and dried from their solutions showed little toxicity in almost all endpoints. Limited *in vitro* toxicity data on these particles are currently available. Both neutralized sulfate and nitrate particles exert little or no effects on inflammatory responses and cell viability^45,46^, and inclusion of strong acids (e.g., sulfuric acid or nitric acid) in particles is proposed to lead to adverse respiratory effects^47,48^. The acid aerosols can increase metal solubility yielding the ROS. Earlier epidemiological studies^26,49–51^ indicate that sulfate has detrimental effects on human health. It is possible that sulfate in the ambient atmosphere reacts with other chemical components, leading to modification of the size and chemistry of the particles, and consequent adverse health effects, in contrast to laboratory-generated sulfate particles^52^. However, the issue of whether sulfate exerts combined effects with other chemical components (e.g., OC and metals) emitted at the same time remains to be established^45,53^.

Fine particles (< 2.5 µm) from Arizona dust^13^ mainly consisting of mineral/soil components showed little toxicity. Additionally, low toxicity was observed for sea spray aerosols from natural seawater based on OP and cell viability analyses (data not shown). However, we observed significant toxicity of resuspended fine dust collected from the roadsides and tunnels at urban sites based on oxidative potential, cell viability, and inflammation endpoints, which may be attributable to carbonaceous species and heavy metals originating from vehicles (engine exhaust, tire wear, and brake pad). Fine particles from carbon black powder (Cabot Inc., USA) exerted a little toxicity, suggesting that carbon black itself would not be so harmful but adsorbed components, such as organics and heavy metals, are the greater contributory factors^54^.

SOA showed substantial toxicity in OP and inflammatory response (IL-8) assays (Fig. 2). Specifically, OP of the SOA produced from photochemical reaction of toluene and NO_x_ under natural sunlight (i.e., toluene SOA) using the UF-APHOR chamber (outdoor smog chamber) was higher than that obtained for other types of SOA (TMB SOA, isoprene SOA, and α-pinene SOA), and was comparable to biomass (hickory wood) burning particles. Toluene is the most abundant volatile organic compound (VOC) emitted from motor vehicles (anthropogenic source)^55,56^ while isoprene SOA is globally the highest VOC from natural sources^57^. The IL-8 response of toluene SOA was higher than TMB SOA. In our study, no cell toxicity (WST-1) was detected for both aromatic and biogenic SOA. The range of exposed mass concentrations of SOA are summarized in Table S2.

**Figure 3:**
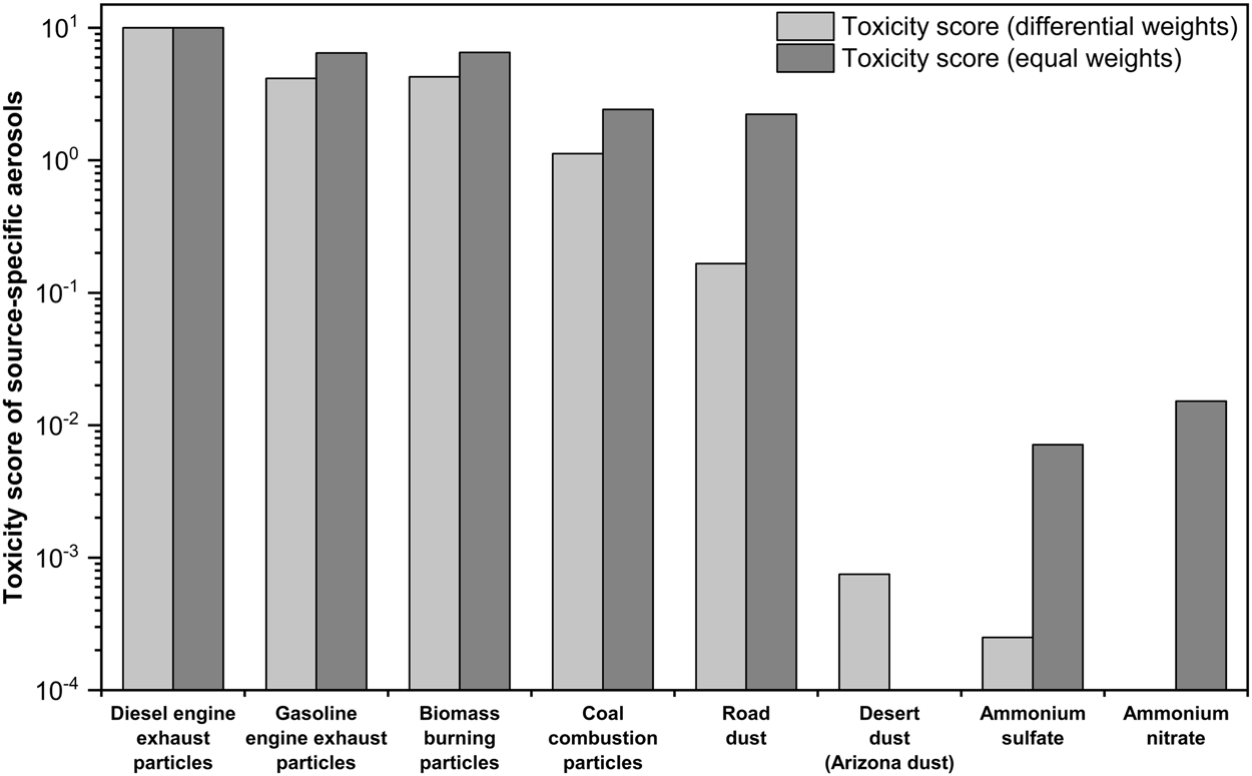
Normalized toxicity scores (0 to 10) for source-specific aerosols with differential weights (CCSD) and equal weights given to endpoints.

As discussed in previous sections, biological and chemical responses for source-specific aerosols varied significantly among toxicological endpoints, making accurate quantification of the representative toxicity difficult. For example, chemical components related to inflammatory responses may be distinct from those associated with bacterial mutagenicity. The relative magnitude of one response would not be feasible to represent toxicity of particles. Average toxicity data obtained for different types of diesel or gasoline engines, biomasses (rice straw and pine stem) and coal combustion temperatures were used to represent diesel engine exhaust, gasoline engine exhaust, biomass burning and coal combustion particles, respectively. A Multiple Attribute Decision Making (MADM) model was applied to derive toxicity scores of source-specific aerosols from various toxicity data. The MADM is to make choice of the best alternative among a set of alternatives with multiple and conflicting attributes^58^. Determination of the weights of attributes is very important in the process of decision making^58^. There are three types of MADM models to determine the weights of attributes (subjective, objective and integrated)^58–60^. In this study, Correlation Coefficient and Standard Deviation (CCSD) method, which is the objective decision making model^58^, was employed to determine the weights of attributes (endpoints) when the outcome (toxicity score) was simultaneously influenced by multiple attributes^58^. In the CCSD method, the endpoint having the highest standard variation among source types had the highest weight (i.e., differential weights were given to endpoints) without any subjective judgement. As resulted from the CCSD, the highest weight was found for cell viability followed by mutagenicity, oxidative potential, inflammatory response, and oxidative stress. The derived weights were multiplied by normalized values of endpoints to determine the toxicity score of source-specific aerosols. The toxicity score was normalized up to 10 (normalized toxicity score of source i = (toxicity score of source i − minimum toxicity score)/(maximum toxicity score − minimum toxicity score) * 10). Source with the highest toxicity has the toxicity score value closest to 10. Multiple toxicological data were integrated into the toxicity score for source-specific aerosols as shown in Fig. 3.

Overall, as shown in Fig. 3, the highest toxicity score was obtained for diesel engine exhaust particles, followed by gasoline engine exhaust particles, biomass burning particles, coal combustion particles, road dust, desert dust, ammonium sulfate, and ammonium nitrate, suggesting that traffic plays the most critical role in enhancing the toxic effects of PM_2.5_. Toluene SOA toxicity based on OP data was comparable to biomass burning particles. In general, the concentration of quinones is negligible in SOA. Jiang *et al*.^57^ reported that alkylperoxides produced from photo-oxidation of precursor hydrocarbons were responsible for the OP_DTT response of the resulting SOA via a non-catalytic reaction mechanism. However, due to the limited number of endpoints for SOA, the SOA data were not included in the CCSD method. In addition to the CCSD method, a linear combination of normalized values of endpoints (i.e., equal weights were given to endpoints) for each source was used to determine the toxicity score (0 to 10) of source-specific aerosols. The toxicity ranking with equal weights was similar to that obtained from the CCSD method with differential weights as shown in Fig. 3. Hereafter, the toxicity score (0 to 10) derived from the CCSD method was used for further calculation.

The aging process of freshly emitted particles in the ambient atmosphere may also modulate toxicity. For instance, aged combustion particles oxidized by ozone are suggested to exacerbate lung injury and inflammation relative to non-oxidized particles^36,61,62^. The effects of atmospheric aging on toxicity of particles require further investigation. The toxicity score for each source could therefore be continuously improved by adding new data sets from *in vitro*, *in vivo*, and epidemiology studies. The toxicity score results should be useful for decision makers to assess contribution of PM_2.5_ sources to human health and to establish PM abatement policies in addition to PM_2.5_ mass concentration.

Typically, source contribution for ambient PM_2.5_ is determined from the measured chemical components using various source apportionment methods^10^. By combining the toxicity scores for source-specific aerosols with the mass fractions of sources in ambient PM_2.5_, toxicity score for ambient PM_2.5_ can be derived. The toxicity score for ambient PM_2.5_ is the summation of values obtained by multiplying toxicity scores for source-specific aerosols by mass fractions of the corresponding sources (toxicity score for ambient PM_2.5_ = toxicity score of source 1 * mass fraction of source 1 + toxicity score of source 2 * mass fraction of source 2 + toxicity score of source 3 * mass fraction of source 3 + toxicity score of source 4 * mass fraction of source 4+ ….). The summation assumes that source-specific toxicity scores are additive.

**Figure 4:**
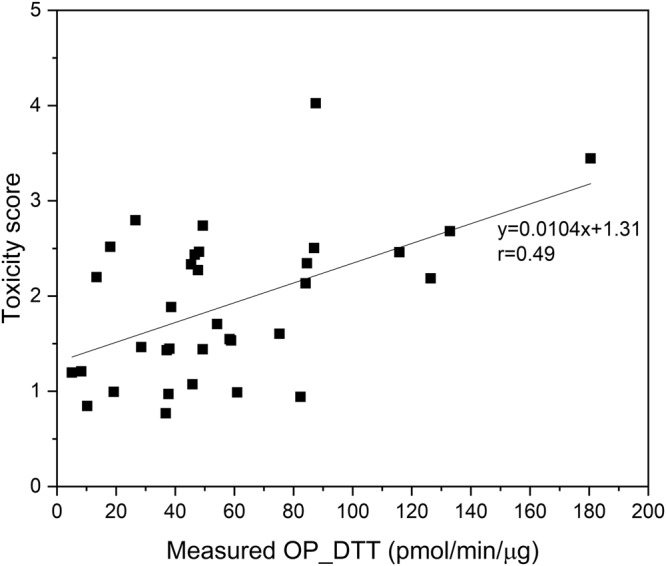
Comparison of daily toxicity score with mass-normalized OP_DTT (pmol/min/µg) for ambient PM_2.5._

We measured the OP_DTT for ambient PM_2.5_ samples. The OP is a good indicator of the oxidizing capability of PM without cell culture and widely used for toxicity testing of ambient aerosols due to its simplicity of application^29^. The oxidizing capability of PM can trigger oxidative stress followed by cell death and can be considered as an additional health metric^29^. The daily toxicity score for ambient PM_2.5_ derived here was compared with the measured OP_DTT (pmol/min/µg) as shown in Fig. 4. It revealed a moderate correlation between the two values (r = 0.49, n = 34). The toxicity score or OP_DTT was found to vary significantly even with similar PM_2.5_ mass concentrations which was attributable to the significant differences in the contributory sources over the experimental days.

**Figure 5:**
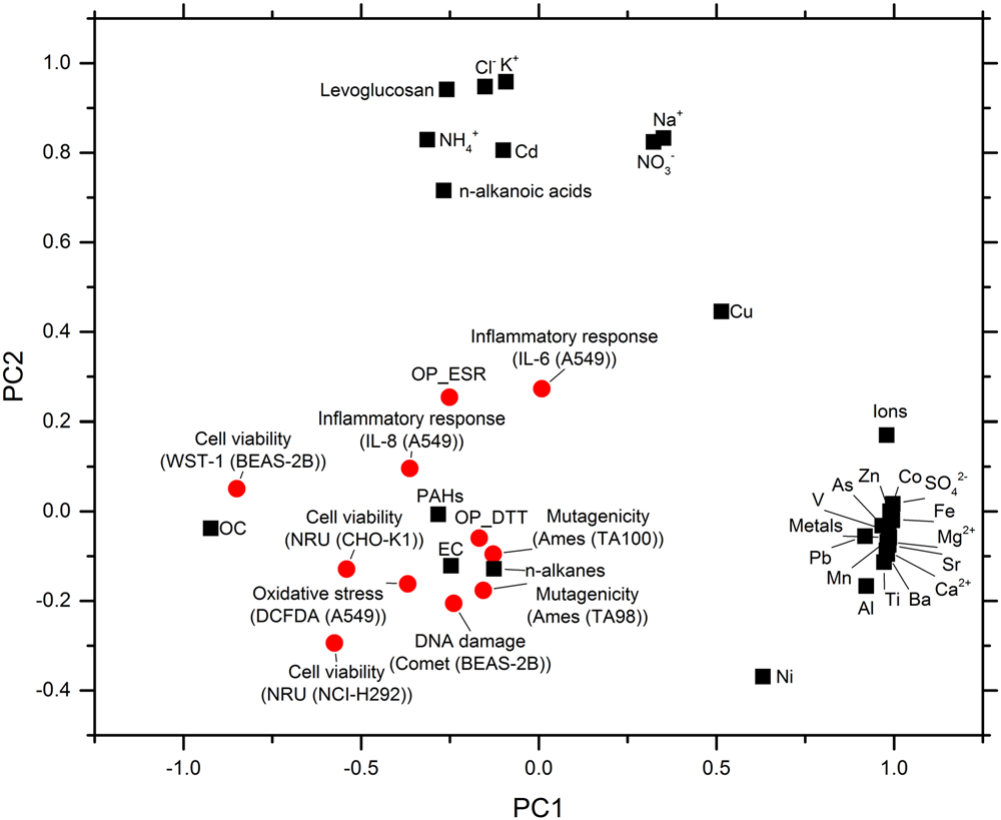
PCA results for relation of chemical and biological responses of source-specific aerosols to their chemical components.

The principal component analysis (PCA) method was applied to relate chemical and biological responses of source-specific aerosols to their chemical components. Details on chemical data for source-specific aerosols are included in Methods and Tables S3, S4, S5, and S6. For PCA, 9 samples (diesel engine exhaust particles (heavy and light duty), gasoline engine exhaust particles, rice straw burning particles, pine stem burning particles, coal combustion particles burned at 550 °C and 1100 °C, dust (tunnel and roadside)) and 30 variables including chemical components (fractions in PM_2.5_) and biological and chemical responses (relative magnitude in each endpoint) were used. The chemical components included 8 ions (ammonium, calcium, magnesium, potassium, sodium, chloride, nitrate, and sulfate), 14 elements (Al, As, Ba, Cd, Co, Cu, Fe, Mn, Ni, Pb, Sr, Ti, V, and Zn), 2 carbonaceous species (OC and EC), and 4 OC classes (n-alkanes, PAHs, alkanoic acids, and sugars (levoglucosan only)). The biological and chemical endpoints were oxidative potential (OP_DTT and OP_ESR), cell viability (NRU (CHO), NRU (H292) and WST-1 (A549)), genotoxicity (mutagenicity and DNA damage), oxidative stress (DCFDA), and inflammatory response (IL-6 and IL-8). As shown in Fig. 5 (loading plot), almost all toxicological endpoints were grouped together with total OC, PAHs, n-alkanes and EC in principal PC1 and PC2. The sum of PC1, PC2 and PC3 explained 78% variance of all data (PC1 = 46%, PC2 = 17% and PC3 = 16%). However, biological responses can be influenced not only by chemical composition but also interaction among chemical species, bioavailability, and oxidation state. Compounding effects between chemical species cannot be fully described by correlation analysis.

## Discussion

Fine particles were generated from distinct combustion (diesel engine, gasoline engine, biomass burning and coal combustion) and non-combustion (road dust, sea spray aerosols, ammonium sulfate, ammonium nitrate and SOA) sources. Measured sizes, chemical components and toxicities of source-specific aerosols varied significantly among the PM_2.5_ source types, suggesting that even at the same mass concentration, the effects of PM_2.5_ on human health are significantly variable. Various biological (cell viability, genotoxicity, oxidative stress and inflammation in respiratory cells) and chemical (oxidative potential) responses to source-specific aerosols were measured and statistically integrated to derive toxicity scores using the MADM model which determines the weights of endpoints when the toxicity score is simultaneously influenced by multiple endpoints. The highest weight was found for cell viability followed by mutagenicity, oxidative potential, inflammatory response, and oxidative stress. The weights for endpoints can be further improved by adding new assays with different cells. Also, the MADM model can be modified by putting more emphasis on specific health endpoints. Diesel engine exhaust particles had the highest toxicity score followed by biomass burning, gasoline engine exhaust, and coal combustion particles. Moreover, different size distributions of aerosols from various sources resulted in variable total and lung deposition efficiencies of particles in the human respiratory system which can be used to estimate lung dose more accurately. The tested method assigned priority to PM_2.5_ sources potentially harmful to human respiratory system. Further incorporation of toxicological data which are related to health effects on cardiovascular system could improve the toxicity score database. *In-vivo* toxicological data for acute toxicity, sub/chronic toxicity, carcinogenicity, and other toxicity can also reinforce the toxicity score.

It is not straightforward to use *in vitro* data (cell viability, genotoxicity, inflammatory response, oxidative potential and oxidative stress) determined in this study to predict health effects (morbidity and mortality) in human populations. In general, *in vitro* data can be used to rank various types of particles in terms of the toxic potential including possible carcinogenicity. Each of the marker we have studied will help to understand the hazard and differential toxicity of various fine particles.

*A priori* knowledge of toxicity of particles produced from various sources obtained here can be linked with source apportionment and exposure level to derive a new health metric for ambient PM_2.5_ in future work. The toxicity potential of various types of particles should be integrated with long term or short term health effects at individual or population level to predict public health impacts with consideration of differential toxicities of fine particles.

## Conclusion

The database for toxicity scores for source-specific aerosols was constructed which can be and used to better understand the complex detrimental health effects caused by different fine particle types of ambient PM_2.5_. By considering differential toxicities of particles although it must be continuously improved, it could be possible to provide information that is more relevant for decision makers to establish PM_2.5_ abatement policies rather than only focusing on PM_2.5_ mass concentration.

## Electronic supplementary material

Supplementary information

## Data Availability

Data from this study are available from the corresponding author upon reasonable request.

## References

[1] Dockery DW (1993). An association between air pollution and mortality in six U.S. cities. New Engl. J. Med..

[2] Pope CA, Bates DV, Raizenne ME (1995). Health effects of particulate air pollution: time for reassessment?. Environ. Health Perspect..

[3] Lelieveld J, Evans JS, Fnais M, Giannadaki D, Pozzer A (2015). The contribution of outdoor air pollution sources to premature mortality on a global scale. Nature.

[4] WHO. *Ambient air pollution: A global assessment of exposure and burden of disease*. World health organization. Available at, http://who.int/phe/publications/air-pollution-global-assessment/en/ (2016).

[5] WHO. *Health relevance of particulate matter from various sources: report on a WHO workshop, Bonn, Germany 26-27 March 2007*. World health organization. Available at, http://www.who.int/iris/handle/10665/107846 (2007).

[6] Cooke RM (2007). A probabilistic characterization of the relationship between fine particulate matter and mortality: elicitation of European experts. Environ. Sci. Technol..

[7] Tuomisto JT, Wilson A, Evans JS, Tainio M (2008). Uncertainty mortality response to airborne fine particulate matter: combining European air pollution experts. Reliab. Eng. Syst. Saf..

[8] Wyzga RE, Rohr AC (2015). Long-term particulate matter exposure: Attributing health effects to individual PM components. J. Air Waste Manage. Assoc..

[9] Health Effects Institute (HEI), *Research report 177, National Particle Component Toxicity (NPACT) Initiative: Integrated Epidemiologic and Toxicologic Studies of the Health Effects of Particulate Matter Components*, Boston, Massachusetts, HEI (2013) 24377209

[10] Institute for Health Metrics and Evaluation (IHME), *Rethinking development and health; findings from the global burden of disease study*. Seattle, WA, IHME. Available at, http://www.healthdata.org/policy-report/rethinking-development-and-health-findings-global-burden-disease-study (2016)

[11] Watson JG (2002). Receptor modeling application framework for particle source apportionment. Chemosphere.

[12] Verma V (2014). Reactive oxygen species associated with water-soluble PM_2.5_ in the southeastern United States: spatiotemporal trends and source apportionment. Atmos. Chem. Phys..

[13] Zheng Y (2012). Sulforaphane prevents pulmonary damage in response to inhaled arsenic by activating the Nrf2-defense response. Toxicol. Appl. Pharmacol..

[14] Febo A, Perrino C (1991). Prediction and experimental evidence for high air concentration of nitrous acid in indoor environments. Atmos. Environ. Part A.

[15] Ng NL (2007). Effect of NOx level on secondary organic aerosol (SOA) formation from the photooxidation of terpenes. Atmos. Chem. Phys..

[16] Wyche KP (2009). Gas phase precursors to anthropogenic secondary organic aerosol: detailed observations of 1,3,5-trimethylbenzene photooxidation. Atmos. Chem. Phys..

[17] Xu L, Kollman MS, Song C, Shilling JE, Ng NL (2014). Effects of NOx on the volatility of secondary organic aerosol from isoprene photooxidation. Environ. Sci. Technol..

[18] Aiken AC (2008). O/C and OM/OC ratios of primary, secondary, and ambient organic aerosols with high-resolution time-of-flight aerosol mass spectrometry. Environ. Sci. Technol..

[19] Birch ME, Cary RA (1996). Elemental carbon-based method for monitoring occupational exposures to particulate diesel exhaust. Aerosol Sci. Technol..

[20] NIOSH. *NIOSH Method 5040Issue 3 (Interim): ElementalCarbon (diesel exhaust)*. National institute for occupational safety and health. Available at, https://www.cdc.gov/niosh/docs/2003-154/pdfs/5040f3.pdf (1999).

[21] Donaldson K (2005). Combustion-derived nanoparticles: A review of their toxicology following inhalation exposure. Part. Fibre Toxicol..

[22] Nel A (2005). Air pollution-related illness: effects of particles. Science.

[23] Cho AK (2005). Redox activity of airborne particulate matter at different sites in the Los Angeles Basin. Environ. Res..

[24] Fang T (2015). PM_2.5_ water-soluble elements in the southeastern United States: automated analytical method development, spatiotemporal distributions, source apportionment, and implications for heath studies. Atmos. Chem. Phys..

[25] Hellack B (2014). Intrinsic hydroxyl radical generation measurements directly from sampled filters as a metric for the oxidative potential of ambient particulate matter. J. Aerosol Sci.

[26] Yang A (2014). Measurement of the oxidative potential of PM_2.5_ and its constituents: The effect of extraction solvent and filter type. Atmos Environ.

[27] Winterbourn CC (2008). Reconciling the chemistry and biology of reactive oxygen species. Nat. Chem. Biol..

[28] Clément B, Merlin G (1995). The contribution of ammonia and alkalinity to landfill leachate toxicity to duckweed. Sci. Total Environ..

[29] Tuet WY (2016). Dose-dependent intracellular reactive oxygen and nitrogen species (ROS/RNS) production from particulate matter exposure: comparison to oxidative potential and chemical composition. Atmos. Environ..

[30] Brenfreund E, Puerner JA (1985). A simple quantitative procedure using monolayer cultures for cytotoxicity assays (HTD/NR-90). J. Tissue Cult. Methods.

[31] Ames BN, McCann J, Yamasaki E (1975). Methods for detecting carcinogens and mutagens with the salmonella/mammalian-microsome mutagenicity test. Mutat. Res.-Environ. Mutag. Related Subj..

[32] Singh NP, McCoy MT, Tice RR, Schneider EL (1988). A simple technique for quantitation of low levels of DNA damage in individual cells. Exp. Cell Res..

[33] Black MJ, Brandt RB (1974). Spectrofluorometric analysis of hydrogen peroxide. Anal. Biochem..

[34] Becker S, Quay J, Soukup J (1991). Cytokine (tumor necrosis factor, IL-6, and IL-8) production by respiratory syncytial virus-infected human alveolar macrophages. J. Immuno..

[35] Singh P (2004). Sample characterization of automobile and forklift diesel exhaust particles and comparative pulmonary toxicity in mice. Environ. Health Perspect..

[36] Delfino RJ, Sioutas C, Malik S (2005). Potential role of ultrafine particles in associations between airborne particle mass and cardiovascular health. Environ. Health Perspect..

[37] Xia T (2004). Quinones and aromatic chemical compounds in particulate matter induce mitochondrial dysfunction: Implications for ultrafine particle toxicity. Environ. Health Perspect..

[38] Antiñolo M, Willis MD, Zhou S, Abbatt JPD (2015). Connecting the oxidation of soot to its redox cycling abilities. Nat. Commun..

[39] International Energy Agency (IEA), *Coal information 2017*, IEA Publication. Available at, https://www.iea.org/statistics/?country=WORLD&year=2015&category=Keyindicators&indicator=CoalConsByType&mode=chart&categoryBrowse=false&dataTable=COALANDPEAT&showDataTable=false (2017).

[40] Makino, H. & Matsuda, H. *Improvement for pulverized coal combustion technology for power generation*. Central Research Institute of Electric Power Industry (CRIEPI), Yokosuka Research Laboratory, Yokosuka, Japan, CRIEPI Rev. **46**. Available at, https://criepi.denken.or.jp/en/energy/research/pdf/Improvement.pdf (2002).

[41] Kim M (2016). Domestic bituminous coal’s calorific value trend analysis (2010–2014) and carbon emission factor development. J. Clim. Change Res..

[42] Maanen JMS (1999). *In vitro* effects of coal fly ashes: Hydroxyl radical generation, iron release, and DNA damage and toxicity in rat lung epithelial cells. Inhal. Toxicol..

[43] Fei WF (2016). The cellular toxicity of PM_2.5_ emitted from coal combustion in human umbilical vein endothelial cells. Biomed. Environ. Sci..

[44] Zhang Y (2008). Characteristics of particulate carbon emissions from real-world Chinese coal combustion. Environ. Sci. Technol..

[45] Schlesinger RB, Cassee F (2003). Atmospheric secondary inorganic particulate matter: the toxicological perspective as a basis for health effects risk assessment. Inhal. Toxicol..

[46] Perrone MG (2010). Seasonal variations in chemical composition and *in vitro* biological effects of fine PM from Milan. Chemosphere.

[47] Grahame TJ, Schlesinger RB (2005). Evaluating the health risk from secondary sulfates in eastern North American regional ambient air particulate matter. Inhal. Toxicol..

[48] Weber RJ, Guo H, Russell AG, Nenes A (2016). High aerosol acidity despite declining atmospheric sulfate concentrations over the past 15 years. Nat. Geosci..

[49] Lippmann M, Ito K, Hwang J-S, Maciejczyk P, Chen L-C (2006). Cardiovascular effects of nickel in ambient air. Environ. Health Perspect..

[50] Cao J, Xu H, Xu Q, Chen B, Kan H (2012). Fine particulate matter constituents and cardiopulmonary mortality in a heavily polluted Chinese city. Environ. Health Perspect..

[51] Kelly, F. J. & Fussell, J. C. Health effects of airborne particles in relation to composition, size and source. In *Airborne Particulate* Matter (eds Hester, R. E. & Harrison, R. M.) 344–382 (Royal Society of Chemistry, Cambridge, 2016).

[52] Kelly FJ, Fussell JC (2012). Size, source and chemical composition as determinants of toxicity attributable to ambient particulate matter. Atmos. Environ..

[53] Cassee FR, Héroux M-E, Gerlofs-Nijland ME, Kelly FJ (2013). Particulate matter beyond mass: recent health evidence on the role of fractions, chemical constituents and sources of emission. Inhal. Toxicol..

[54] Odum JR, Jungkamp TPW, Griffin RJ, Flagan RC, Seinfeld JH (1997). The atmospheric aerosol-forming potential of whole gasoline vapor. Science.

[55] Zhang X (2014). Influence of vapor wall loss in laboratory chambers on yields of secondary organic aerosol. Proc. Natl. Acad. Sci. USA.

[56] Claeys M (2004). Formation of secondary organic aerosols through photooxidation of isoprene. Science.

[57] Jiang H, Jang M, Yu Z (2017). Dithiothreitol activity by particulate oxidizers of SOA produced from photooxidation of hydrocarbons under varied NO_x_ levels. Atmos. Chem. Phys..

[58] Wang YM, Luo Y (2010). Integration of correlations with standard deviations for determining attribute weights in multiple attribute decision making. Math. Comput. Modelling.

[59] Zhang Q, Chen JCH, He Y-Q, Ma J, Zhou D-N (2003). Multiple attribute descision making: approach integrating subjective and objective information. Int. J. Manuf. Technol. Manage..

[60] Liu H, Kong F (2005). A new MADM algorithm based on fuzzy subjective and objective integrated weights. Int. J. Inf. Syst. Sci..

[61] McWhinney RD, Gao SS, Zhou S, Abbatt JPD (2011). Evaluation of the effects of ozone oxidation on redox-cycling activity of two-stroke engine exhaust particles. Environ. Sci. Technol..

[62] Asgharian B, Hofmann W, Bergmann R (2001). Particle deposition in a multiple-path model of the human lung. Aerosol Sci. Technol..

