# Supplementary material for: Differential toxicities of fine particulate matters from various sources
Source: Sci Rep. 2018 Nov 19;8:17007. doi: 10.1038/s41598-018-35398-0 (PMC6242998; doi:10.1038/s41598-018-35398-0)
Supplement: Supplementary file 1 — Supplementary information [file 41598_2018_35398_MOESM1_ESM.docx]

**Supplementary Information**

**Differential toxicities of fine particulate matters from various sources**

Minhan Park^1^, Hung Soo Joo^1,2^, Kwangyul Lee^1^, Myoseon Jang^3^, Sang Don Kim^1^, Injeong Kim^1^, Lucille Joanna S. Borlaza^1^, Heungbin Lim^4^, Hanjae Shin^5^, Kyu Hyuck Chung^6^, Yoon-Hyeong Choi^7^, Sun Gu Park^7^, Min-Suk Bae^8^, Jiyi Lee^9^, Hangyul Song^1^, and Kihong Park^1,*^

^1^School of Earth Sciences and Environmental Engineering, Gwangju Institute of Science and Technology (GIST), Gwangju, Republic of Korea. ^2^Department of Environmental Engineering, Anyang University, Anyang, Republic of Korea. ^3^Department of Environmental and Global Health, University of Florida, Gainesville, FL, USA. ^4^Department of Industrial Plant Science & Technology, Chungbuk National University, Cheongju, Republic of Korea. ^5^R&D Headquarter, KT&G, Daejeon, Republic of Korea. ^6^School of Pharmacy, Sungkyunkwan University, Suwon, Republic of Korea. ^7^Department of Preventive Medicine, Gachon University Graduate School of Medicine, Incheon, Republic of Korea. ^8^Department of Environmental Engineering, Mokpo National University, Muan, Republic of Korea. ^9^ Department of Environmental Science and Engineering, Ewha Womans University, Seoul, Republic of Korea.

**Corresponding Author:**

Kihong Park, School of Earth Sciences and Environmental Engineering, Gwangju Institute of Science and Technology (GIST), Gwangju 61005, Republic of Korea, +82-62-715-3279, kpark@gist.ac.kr

Table S1. Results for chemical and biological responses for fine particles (PM_2.5_) produced from different sources

|  |  |  | **Biomass burning particles** | | **Coal combustion particles** | | **Diesel engine exhaust particles** | | **Gasoline engine exhaust particles** | **Road dust** | | **Arizona dust** | **Black carbon** | **Ammonium sulfate** | **Ammonium nitrate** |
| --- | --- | --- | --- | --- | --- | --- | --- | --- | --- | --- | --- | --- | --- | --- | --- |
| **Endpoint** | **Assay** | **Unit** | **Rice straw** | **Pine stem** | **1100°C^a^** | **550°C^a^** | **2800 cc^b^** | **498 cc^b^** | **50 cc^b^** | **Tunnel** | **Roadside** |  |  |  |  |
| Oxidative potential | OP_DTT | pmol/min/μg^c^ | 53.1±20.9 | 7.7±3.7 | 0.2±0.6 | 26.2±35.5 | 297.0±450.4 | 3.1±1.7 | 2.2 | 13.4±22.9 | 8.3±8.5 | 0.1 | 0.3±0.1 | 0.05±0.06 | 0.02±0.03 |
|  | OP_ESR | AU/μg^c^ | 20.2±21.0 | 1.3±1.3 | 1.9±2.2 | 8.6±14.2 | 0.36 | NS | 11.5 | 3.7±3.4 | 4.1±5.4 | NS | NS | 0.08±0.09 | 0.17±0.17 |
| Cell viability | NRU (CHO) | μg/ml^d^ | 40.2±14.3 | 40.7±6.7 | NS | 67.0±17.7 | 65.0±13.4 | 15.4±1.3 | 56.0±6.2 | NS | NS | NS | NS | NS | NS |
|  | NRU (H292) | μg/ml^d^ | 73.1±5.4 | 57.5±8.8 | NS | 125.0±10.1 | 46.4±1.9 | 12.8±10.3 | 32.4±8.1 | 134.7±17.0 | NS | NS | NS | NS | NS |
|  | WST-1 | μg/ml^d^ | 554.7±5.3 | 332.8±16.8 | NS | 591.6±21.4 | 358.4±43.5 | 393.0±11.1 | 331.9±14.0 | NS | NS | NS | NS | NS | NS |
| Genotoxicity | Mutagenicity (Ames (TA98)) | revertants/mg^e^ | 649.7±199.2 | 891.9±174.4 | NS | NS | 44247.4±648.2 | 680.7±10.7 | 10848.9±1078.7 | NS | NS | NS | NS | NS | NS |
|  | Mutagenicity (Ames (TA100)) | revertants/mg^e^ | 1032.2±100.6 | NS | NS | NS | 28965.1±1756.7 | NS | 12106.5±576.2 | NS | NS | NS | NS | NS | NS |
|  | Mutagenicity (Ames (TA1535)) | revertants/mg^e^ | 34.5±31.02 | 92.7±10.2 | NS | NS | 1291.5±432.7 | NS | NS | 26.8±3.5 | NS | NS | NS | NS | NS |
|  | Mutagenicity (Ames (TA1537)) | revertants/mg^e^ | 215.7±159.3 | 263.6±133.3 | NS | NS | 8839.5±659.2 | 69.2±39.4 | 2623.2±196.6 | 42.2±13.9 | NS | NS | NS | NS | NS |
|  | DNA damage (Comet) | SCDI^f^ | 0.02 | 0.06 | NS | 0.05 | 0.02 | 0.02 | 0.4 | NS | 0.009 | - | - | - | - |
| Oxidative stress | ROS production (DCFDA) | Fold of control^g^ | 1.1±0.02 | 1.1±0.1 | NS | NS | 3.8±1.0 | 3.1±0.02 | 1.1±0.02 | 1.1±0.2 | 1.1±0.2 | NS | NS | NS | NS |
| Inflammatory response | IL-6 | Fold of control/μg^h^ | NS | 1.8±0.1 | NS | NS | 1.0±0.1 | 1.4±0.3 | 0.8±0.5 | 0.2±0.004 | NS | NS | NS | NS | NS |
|  | IL-8 | Fold of control/μg^h^ | NS | 0.5±0.02 | NS | NS | 0.3±0.01 | 0.4±0.06 | NS | NS | NS | NS | NS | NS | NS |
| Values represent mean ± SD  NS; Non-significant  ^a^ Coal combustion temperature  ^b^ Engine displacement  ^c^ OP activity normalized by PM_2.5_ mass  ^d^ EC_50_; half-maximal effective concentration  ^e^ Specific activity; the number of revertant colonies per PM_2.5_ mass  ^f^ SCDI (significant concentration-dependent induction factor); the summation of fold change over control in the concentration range with significant dose-response relationship  ^g^ Fluorescence intensity; the fold change over control at 60 μg/ml dose concentration  ^h^ The maximum cytokine production; the maximum fold change over control from dose-response curve | | | | | | | | | | | | | | | |

Table S2. SOA produced using the outdoor chamber^a^

| **Precursor hydrocarbon (HC)** | **Initial HC (ppb)** | **Initial HC/NO_x_**  **(ppbC/ppb)** | **ΔHC^b^**  **(ppb)** | **SOA yield at maximum^c^** |
| --- | --- | --- | --- | --- |
| Toluene | 622-939 | 16-24 | 517-549 | 7.1-9.1 |
| Toluene | 640-691 | 5-6 | 421-541 | 8.1-14.3 |
| TMB | 583-657 | 19-34 | 414-542 | 5.7-7.8 |
| TMB | 589-613 | 5-6 | 548-613 | 5.6-6.7 |
| Isoprene | 2718-3173 | 27-32 | 2718-3173 | 1.2-1.4 |
| Isoprene | 2525-3060 | 5 | 2525-3060 | 3.3-47 |
| α-pinene | 257-323 | 36 | 323 | 36.1 |
| α-pinene | 318 | 5 | 318 | 14.5 |

^a^ Outdoor chamber experiments were repeated to analyze chemical compositions and ensure repeatability, thus the experimental conditions and data were in the form of range. All SOA were produced in the absence of inorganic seed aerosol.

^b^ ΔHC is the consumption of HC when the SOA concentration reached a maximum during the aerosol collection.

^c^ The SOA yield is estimated from the ratio of organic mass formed to HC consumption (Odum et al.^1^). SOA mass is estimated using SMPS and the density of SOA aerosol.

Table S3. A summary for fractions (%) of chemical components (ions, OC, EC, and elements) in PM_2.5_ produced from different sources

|  | **Biomass burning particles** | | **Coal combustion particles** | | | **Diesel engine exhaust particles** | | | **Gasoline engine exhaust particles** | **Road dust** | | **Sea spray aerosols** |
| --- | --- | --- | --- | --- | --- | --- | --- | --- | --- | --- | --- | --- |
| **Chemical components** | **Rice straw** | **Pine stem** | **1100°C^a^** | **900°C^a^** | **550°C^a^** | **7620 cc^b^** | **2800 cc^b^** | **498 cc^b^** | **50 cc^b^** | **Tunnel** | **Roadside** | **Seawater** |
| Ions | 7.0 | 6.9 | 38.4 | 76.5 | 3.1 | 3.8 | 2.9 | 0.2 | 0.8 | 3.3 | 0.5 | 87.1 |
| OC | 67.2 | 55.9 | 1.0 | 0.08 | 67.4 | 76.7 | 69.4 | 70.7 | 64.6 | 26.9 | 27.4 | 1.5 |
| EC | 2.0 | 10.8 | <0.001 | <0.001 | 4.3 | 2.2 | 3.8 | 1.3 | 12.1 | 4.9 | 4.7 | 0.006 |
| Elements | 0.3 | 0.6 | 14.9 | 11.0 | 2.5 | 0.2 | 1.0 | 0.03 | 0.04 | 11.4 | 3.3 | 0.05 |
| ^a^ Coal combustion temperature  ^b^ Engine displacement | | | | | | | | | | | | |

Table S4. A summary for fractions (%) of organic compounds in PM_2.5_ produced from different sources

|  | **Biomass burning particles** | | **Coal combustion particles** | | | **Diesel exhaust particles** | | | **Gasoline engine exhaust particles** |
| --- | --- | --- | --- | --- | --- | --- | --- | --- | --- |
| **Organic compounds** | **Rice straw** | **Pine stem** | **1100°C^a^** | **900°C^a^** | **550 °C^a^** | **7620 cc^b^** | **2800 cc^b^** | **498 cc^b^** | **50 cc^b^** |
| PAHs | 0.2 | 1.4 | 0.003 | ND | 0.6 | ND | 0.3 | 0.07 | 3.3 |
| n-alkanes | 1.1 | 0.2 | 0.01 | 0.003 | 0.04 | 1.2 | 38.7 | 1.7 | 0.1 |
| cyclo-alkanes | ND | ND | - | - | - | - | - | - | - |
| hopanes and steranes | ND | ND | ND | ND | 0.02 | - | - | - | - |
| hydroxy-PAHs | - | - | ND | ND | ND | - | - | - | - |
| n-alkanoic acids | 8.8 | 2.5 | 0.01 | 0.03 | ND | - | 2.1 | 0.2 | 0.2 |
| resin acids | <0.001 | 3.6 | - | - | - | - | - | - | - |
| benzoic acids | 0.006 | 0.04 | - | - | - | - | - | - | - |
| levoglucosan | 3.2 | 4.3 | 0.003 | ND | ND | - | 0.02 | ND | ND |
| dicarboxylic acids | 0.4 | 0.04 | ND | 0.002 | ND | - | - | - | - |
| ND; Not detected  ^a^ Coal combustion temperature  ^b^ Engine displacement | | | | | | | | | |

Table S5. A summary for fractions (%) of ionic species in PM_2.5_ produced from different sources

|  | **Biomass burning particles** | | **Coal combustion particles** | | | **Diesel engine exhaust particles** | | | **Gasoline engine exhaust particles** | **Road dust** | | **Sea spray aerosols** |
| --- | --- | --- | --- | --- | --- | --- | --- | --- | --- | --- | --- | --- |
| **Ionic species** | **Rice straw** | **Pine stem** | **1100°C^a^** | **900°C^a^** | **550°C^a^** | **7620 cc^b^** | **2800 cc^b^** | **498 cc^b^** | **50 cc^b^** | **Tunnel** | **Roadside** | **Seawater** |
| SO_4_^2-^ | 1.6 | 1.6 | 32.3 | 59.7 | 1.9 | 0.4 | 2.1 | 0.005 | 0.02 | 0.8 | 0.09 | 10.1 |
| NO_3_^-^ | 0.7 | 0.9 | 0.7 | ND | 0.03 | 2.2 | 0.04 | 0.2 | 0.5 | 0.1 | 0.01 | 1.6 |
| Cl^-^ | 2.2 | 1.8 | 0.3 | 0.006 | ND | 0.4 | 0.04 | <0.001 | 0.008 | 0.08 | 0.006 | 44.6 |
| Na^+^ | 1.1 | 0.7 | 0.8 | 10.4 | 0.2 | 0.2 | 0.04 | 0.002 | 0.01 | 0.2 | 0.01 | 25.5 |
| NH_4_^+^ | 0.5 | 0.6 | 0.02 | ND | 0.02 | 0.5 | 0.5 | 0.002 | 0.03 | ND | 0.004 | 0.008 |
| K^+^ | 0.7 | 1.1 | 0.2 | 0.8 | 0.02 | 0.04 | 0.006 | 0.004 | 0.008 | 0.6 | 0.03 | 0.9 |
| Mg^2+^ | 0.02 | 0.04 | 0.8 | 2.1 | 0.2 | 0.009 | 0.05 | 0.002 | 0.01 | 0.04 | 0.01 | 3.2 |
| Ca^2+^ | 0.08 | 0.2 | 3.3 | 3.6 | 0.7 | 0.1 | 0.1 | 0.02 | 0.2 | 1.5 | 0.3 | 1.2 |
| ND: Not detected  ^a^ Coal combustion temperature  ^b^ Engine displacement | | | | | | | | | | | | |

Table S6. A summary for fractions of elements (%) in PM_2.5_ produced from different sources

|  | **Biomass burning particles** | | **Coal combustion particles** | | | **Diesel exhaust particles** | | | **Gasoline engine exhaust particles** | **Road dust** | | **Sea spray aerosols** |
| --- | --- | --- | --- | --- | --- | --- | --- | --- | --- | --- | --- | --- |
| **Elements** | **Rice straw** | **Pine stem** | **1100°C^a^** | **900°C^a^** | **550°C^a^** | **7620 cc^b^** | **2800 cc^b^** | **498 cc^b^** | **50 cc^b^** | **Tunnel** | **Roadside** | **Seawater** |
| Al | 0.05 | 0.07 | 3.5 | 2.2 | 1.4 | 0.1 | 0.1 | 0.01 | 0.02 | 4.9 | 1.3 | 0.02 |
| Ti | - | - | 0.1 | 0.04 | 0.02 | 0.006 | 0.003 | ND | ND | 0.2 | 0.06 | ND |
| V | <0.001 | <0.001 | 0.01 | 0.002 | 0.002 | <0.001 | <0.001 | <0.001 | <0.001 | 0.006 | 0.002 | <0.001 |
| Mn | <0.001 | 0.006 | 0.2 | 0.2 | 0.03 | 0.001 | 0.002 | <0.001 | 0.002 | 0.08 | 0.03 | <0.001 |
| Fe | 0.2 | 0.5 | 10.2 | 7.7 | 0.9 | 0.05 | 0.8 | 0.007 | 0.006 | 3.5 | 1.0 | 0.008 |
| Ni | - | - | 0.03 | 0.01 | 0.03 | 0.002 | 0.002 | - | 0.001 | 0.01 | 0.005 | - |
| Cu | 0.01 | 0.02 | 0.02 | 0.006 | 0.02 | 0.003 | 0.005 | 0.001 | 0.001 | 0.03 | 0.009 | <0.001 |
| Zn | 0.02 | 0.01 | 0.4 | 0.7 | 0.02 | 0.03 | 0.06 | 0.01 | 0.010 | 2.4 | 0.9 | 0.004 |
| As | <0.001 | <0.001 | 0.004 | 0.005 | 0.001 | <0.001 | <0.001 | - | ND | 0.003 | 0.001 | - |
| Sr | <0.001 | <0.001 | 0.2 | 0.06 | 0.03 | <0.001 | <0.001 | <0.001 | <0.001 | 0.03 | 0.009 | 0.02 |
| Cd | <0.001 | <0.001 | <0.001 | <0.001 | <0.001 | <0.001 | <0.001 | - | - | <0.001 | <0.001 | <0.001 |
| Ba | <0.001 | <0.001 | 0.2 | 0.07 | 0.03 | 0.005 | 0.004 | <0.001 | 0.001 | 0.1 | 0.06 | <0.001 |
| Pb | <0.001 | 0.003 | 0.02 | 0.004 | 0.006 | <0.001 | 0.002 |  | <0.001 | 0.03 | 0.01 | <0.001 |
| Co | <0.001 | <0.001 | 0.01 | 0.01 | 0.001 | <0.001 | <0.001 | <0.001 | <0.001 | - | - | <0.001 |
| ND: Not detected  ^a^ Coal burning temperature  ^b^ Engine displacement | | | | | | | | | | | | |

Table S7. A summary for diesel and gasoline engines tested in this study

| **Engine type** | **Diesel engine**  **(heavy duty)** | **Diesel engine**  **(light duty)** | **Diesel engine**  **(generator)** | **Gasoline engine (generator)** |
| --- | --- | --- | --- | --- |
| Engine model | DL08S | 4JB1 | 192FC | GXH50 |
| Manufacturer | Doosan Infracore,  (Korea) | Isuzu motors  (Japan) | Hi-earns  (China) | Honda  (Japan) |
| Engine type | 4-cycle, 6-cylinder | 4-cylcle, 4-cylinder | 4-cycle, 1-cylinder | 4-cycle, 1-cylinder |
| Engine displacement (cc) | 7620 | 2800 | 498 | 50 |
| Operating torque/maximum torque (Nm) | 10/1422 | 10/179 | - | - |
| Operating engine speed (rpm) | 1500 | 1500 | 3000 | 5500 |
| Operating power output/maximum power output (kw) | 2.1/221 | 2.1/28 | 8/8 | 1/1 |

Table S8. A summary for OP and in-vitro toxicity test conditions used in this study

| **Endpoints** | **Assay** | **Cell types** | **Positive control** | **Negative control** | **Dose concentration**  **(μg/ml)** | **Exposure time** |
| --- | --- | --- | --- | --- | --- | --- |
| Oxidative potential | OP_DTT | - | Phenanthrenequinone (PQN) | DI water | 25-2591 | 5, 15, 25, 35,and 45 min |
| Oxidative potential | OP_ESR | - | - | DI water | 25-2591 | < 1 h |
| Cell viability | NRU | CHO-K1,  NCI-H292 | - | Culture medium containing 1.5% DMSO | 1-150 | 24 h |
| Cell viability | WST-1 | BEAS-2B | - | Culture medium | 10-500 | 24 h |
| Cell viability | WST-1 | SAEC | - | Blank filter extract in DI water | 3.5-17.5 | 24 h |
| Genotoxicity  (Mutagenicity) | Ames test | *Salmonella typhimurium*.  (TA98, TA100, TA1535, TA1537) | 2-aminoanthracene | Culture medium containing 1.5% DMSO | 0.1-1000 | 48 h |
| Genotoxicity  (DNA damage) | Comet | BEAS-2B | - | Culture medium | 62.5-500 | 24 h |
| Oxidative stress | DCFDA | A549 | Tert-butyl hydrogen peroxide (TBHP) | Culture medium containing 1.5% DMSO | 15-150 | 24 h |
| Inflammatory response | IL-6, IL-8 | A549 | - | Culture medium containing 1.5% DMSO | 2.5-100 | 24 h |
| Inflammatory response | IL-6, IL-8 | SAEC | - | Blank filter extract in  DI water | 3.5-17.5 | 24 h |
|  | | | | | |  |

**Methods for measuring chemical and biological toxicity of source-specific aerosols**

**Dithiothreitol (DTT) assay**

To perform OP analysis, water-soluble PM_2.5_ was extracted by subjecting filter samples to sonication and shaking for 1 hour submerged in a 5 ml of DI water. The extracted solution was filtered by 0.45 μm pore size PTFE syringe filter (Advantec, Japan) to eliminate insoluble components. The filtered extract was used for both OP_DTT and OP_ESR measurements.

For the OP_DTT analysis, a 3 ml of PM_2.5_ extract was mixed with a 14.5 ml DI water, a 5 ml of 50 mM potassium phosphate buffer (pH = 7.4), and a 2.5 ml of 5 mM DTT in an amber vial. The vial was subjected to shaking and incubation at a specific time ranging from 5, 15, 25, 35 and 45 minutes by shaking incubator at 37°C, 200 rpm. After incubation, 1 ml of 1% (w/v) trichloroacetic acid (TCA) quenching agent and 1 ml of 5,5-dithiobis- (2-nitrobenzoic acid) (DTNB) in another amber vial were successively added to the vial. Then, a 4 ml of 80 mM Tris buffer (pH = 8.9) solution with a 4 mM ethylenediaminetetraacetate (EDTA) was added to the vial to stabilize the mixture. The reaction of the DTT with the DTNB produced a yellow chromophore (5-mercapto-2-nitrobenzoic acid or TNB) which was quantified by the UV-VIS spectrophotometer (Spectramax M2, Molecular Devices LCC). The DTT consumption rate was calculated using a linear regression of plotted absorbance against time (pmol/min).

For the OP_ESR analysis, a 0.1 ml of PM_2.5_ extract was mixed with a 1 ml of 0.5 M H_2_O_2_, a 2 ml of 0.05 M DMPO, and a 1 ml of DI water. The solution was incubated at 37°C and 200 rpm for 15 min in shaker. After incubation, the solution was subjected to vortexing for 20 secs before analysis using the ESR spectrometer (JES-FA200, JEOL Ltd., Japan). The OP_ESR value was calculated by averaging the total amplitudes of DMPO-OH quartets. The OP_DTT and OP_ESR activity were calculated by multiplying the reciprocal of absolute PM mass by the OP_DTT consumption rate and OP_DTT value, respectively.

**Cells and cell culture**

The NCI-H292 and CHO-K1 cell lines (Korea Cell Line Bank, Korea) were used to measure the cell viability by the NRU assay^2^, the A549 cell line (Korea Cell Line Bank, Korea) was used to measure cell viability by the WST-1 assay, oxidative stress, and inflammatory response (IL-6 and IL-8), and the BEAS-2B cell lines (American Type Culture Collection, USA) were used to measure the DNA damage (Comet assay)^3^. The small airway epithelial cells (SAEC) were also used to measure inflammatory response (IL-6 and IL-8) for the SOAs. The 4 types of *salmonella typhimurium* strains (TA98, TA100, TA1535, and TA1537) were used to measure mutagenicity (Ames test)^4^. The CHO-K1 and BEAS-2B cells were grown in in Ham’s F-12 medium (Gibco, UK) and Dulbecco modified eagle medium (DMEM) (Gibco, UK), respectively, and the NCI-H292, A549, and SAEC cells were grown in Roswell Park Memorial Institute (RPMI) 1640 medium (Gibco, UK) with 10% Fatal Bovine Serum (FBS) (Gibco, UK), 100 units/mL of penicillin and 100 µg/mL of streptomycin (Gibco, UK) in the incubator of humidified atmosphere (5% CO_2_ and 95% air) at 37°C.

**Neutral red uptake (NRU) assay**

Cytotoxicity was measured by a Neutral red uptake (NRU) assay. The filter sample was extracted in dimethyl sulfoxide (DMSO) for 1 h by sonicator and shaking incubator. The PM_2.5_ extract was filtered by 0.22 μm PTFE syringe filter and stored at -20 °C until use. The CHO-K1 and NCI-H292 cell lines were seeded on 96-well plates at a density 1.0 x 10^4^ cells/well and 1.5 x 10^4^ cells/well, respectively, with culture medium. The 96-well plates were placed on a humidified atmosphere at 37°C and 5% CO_2_ for 24h. The culture medium was then removed and the diluted PM_2.5_ extract or culture medium (negative control) was treated in each well for 24h. After 24h exposure, the neutral red (NR) solution (50 µg/mL in culture medium) was treated for 2h. After the neutral red solution was aspirated, wash-fix solution (1% (v/v) formalin) was added to each well. The wash-fix solution was then removed within 1 min and a mixture of 1% acetic acid-50% ethanol was added to each well to extract the NR solution from cells at room temperature for 15 min. Optical density was measured at 540 nm by a microplate reader (SpectraMax M2, Molecular Devices, USA). The EC_50_ value (dose of 50% inhibition on cell growth) was calculated by the regression of the dose-response curve.

**Water soluble tetrazolium salt (WST-1) assay**

Water soluble tetrazolium salt (WST-1) assay was used to measure the cell viability. The filter sample was extracted by dichloromethane (DCM) for 4 h with sonication. DCM was evaporated and completely dried out by rotary evaporator (Eyela 116810, Tokyo Rikakikai Co., LTD., Japan) and N_2_ blowing. The extract was dissolved in DMSO and adjusted to the final concentration of 50 mg PM_2.5_/ml. And then the extracts were filtered by 0.22 μm PTFE syringe filter and stored at -20 °C until use. The BEAS-2B cells were seeded onto 96-well plates at a density of 5×10^3^ cells/well. After 24 h, the PM_2.5_ extract or culture medium (negative control) were treated for 24 h. To measure cell viability, 10 μL of WST-1 reagent (MK400, Takara, Japan) was added to each well after the removal of 100 μL culture medium. After 3 hours of incubation, the light absorbance at 440 nm and 690 nm were measured by a microplate reader (Versamax, Molecular Devices, USA). Cell viability was calculated as a percentage relative to that of the control cells. The EC_50_ was calculated by the regression of the dose-response curve.

**The Ames *salmonella*/microsome mutagenicity (Ames) assay**

Mutagenicity was evaluated according to method described by the Organization for Economic Cooperation and Development (OECD) guideline^5^. A filter sample was extracted in dimethyl sulfoxide (DMSO) with 1 hour sonication and shaking. The PM_2.5_ extract was filtered by 0.22 μm PTFE syringe filter and then stored at -20 ^o^C until use. The *Salmonella typhimurium* strains was used for the determination of mutagenicity (Ames test). The *Salmonella typhimurium* strains was grown with 2.5% nutrient liquid at 37^o^C in a shaking incubator maintained at a 80 rpm for 10 h. Approximately 1 x 10^9^ bacteria/ml suspended in 100 μL of culture medium, and 100 μL of PM_2.5_ extract was diluted in culture medium. The 500 μL of S-9 mixture (5% v/v) were added to 2mL of top agar containing histidine and biotin. The mixture was mixed and spread on minimal glucose agar plates. After the top agar was hardened, the plates were incubated at 37 ^o^C under darkness for 48h. The number of revertant colonies were manually counted and averaged of triplicates. The specific activity was calculated as the slope (revertant colonies/mg of PM_2.5_) of linear portion of dose-response curve. DMSO without PM_2.5_ extract was used for negative control.

**Comet assay**

The comet assay was performed as described by the Singh et al.^3^. The methods for the sample preparation including PM_2.5_ extraction were the same as the WST-1’s. The BEAS-2B cells were seeded on 6-well plates at a density of 2×10^5^ cells/well. After 24h of incubation, the cultured cells were exposed to the diluted PM_2.5_ extract or culture medium (negative control) for 24 h. The treated cells were resuspended in 0.7% low melting point agar. The 160-μL aliquot of each cell suspension was spread onto a pre-coated glass slide and covered with a cover glass after which the slide was incubated for 1 h at 4˚C. Then, cells were lysed in pH 10 lysis solution at 4^o^C for 1h. The lysed cells were allowed to unwind for 30 min in electrophoresis buffer before electrophoresis for 30 min at 25 V on ice. The gels were neutralized with 0.4 M Tris-HCl (pH 7.5) twice for 5 min and stained with ethidium bromide (2 μg/mL). The DNA migration was assessed by automatic image analysis software (Komet version 5.0, Kinetic Imaging Ltd., UK). The Olive tail moment (OTM; tail distance×percentage of DNA in the tail) was used to quantify DNA damage based on random scoring of 50 nuclei per slide. To compare genotoxicity of samples, the significant concentration-dependent induction factor (SCDI) method was used^6^. The equation of SCDI is as following;

$$\mathbf{SCDI=}\sum_{\mathbf{i=1}}^{\mathbf{n}^{\mathbf{*}}} \mathbf{IF}_{\mathbf{i}}^{\mathbf{*}}\mathbf{/}\mathbf{c}_{\mathbf{i}}^{\mathbf{*}}$$

where IF_i_ stands for the induction factor of the concentration, Ci was the concentration i, and n was the number of doses. For SCDI, IF_i_* and C_i_* represents induction factor and concentration that showed significant difference between control and treated group.

**2’,7’ –dichlorofluorescin diacetate (DCFDA) assay**

The intracellular ROS which can be an indicator for oxidative stress was measured by 2’,7’ –dichlorofluorescin diacetate (DCFDA) assay kit (Abcam, UK). The filter sample was extracted in dimethyl sulfoxide (DMSO) for 1 h by sonicator and shaking incubator. The PM_2.5_ extract was filtered by 0.22 μm PTFE syringe filter and stored at -20 °C until use. The A549 cell lines were seeded on 96-well plates at a density 2.5 x 10^4^ cell per well with culture medium for 24h. The cells were stained with 25 µM DCFDA for 45min at 37 °C in dark. The DCFDA solution was removed and diluted PM_2.5_ extract or culture medium (negative control) was replaced in each well and treated for 4h. The fluorescence was measured at maximum excitation and emission spectra of 495 nm and 529 nm, respectively, using a microplate reader (SpectraMax M2, Molecular Devices, USA). The intracellular ROS value was calculated as fold of control at 60 µg/ml dose concentration from dose-response curve.

**Enzyme-linked immunosorbent (ELISA) assay**

Inflammatory responses were measured by commercial enzyme-linked immunosorbent assay (ELISA) kits for IL-6 (abcam, UK) and IL-8 (abcam, UK). The filter sample was extracted in dimethyl sulfoxide (DMSO) for 1 h by sonicator and shaking incubator. The PM_2.5_ extract was filtered by 0.22 μm PTFE syringe filter and stored at -20 °C until use. The A549 cells were seeded on 6-well plates at a density of 2.5×10^5^ cells/well and incubated for 24h. The cultured cells were then exposed to the diluted PM_2.5_ extract or culture medium (negative control) for 24 h. After 24 h, the culture medium was centrifuged at 2500 rpm for 10 min. Then, supernatants were added to 96 well plate on which capture antibody was pre-coated, and the plate was incubated for 2 h. After 2 h, the supernatants were replaced with detection antibody and incubated for 2 h. Then, the detection antibody was removed and streptavidin-HRP was added. After 20 min, the substrate solution of 3,3',5,5'- tetramethyl benzidine was added to each well containing streptavidin-HRP and then stop solution was added. The observance was measured at 450 nm using a microplate reader (SpectraMax M2, Molecular Devices, USA). The cytokine level was calculated as the slope (fold of control/µg PM_2.5_) of the linear portion of dose-response curve.

**Gene expression analysis (IL-6 and IL-8)**

For the SOA samples, the gene expression of IL-8 and IL-6 was performed by human SAECs as described in Jiang et al.^7^. Briefly, SAECs were seeded in 24-well plates and then exposed by SOAs which obtained by a particle-into-liquid sampler (PILS). After 24 h, RNA extraction was performed using RNA STAT-60 (Tel-Test Inc., USA), chloroform and GlycoBlue (Ambion, USA). After extraction, RNA extract were inverted and kept at -20 ℃ for overnight. The RNA pellet was washed with 75% (v/v) ethanol and repeated after centrifugation at 14000 rpm for 45 min (4 °C). After washing, RNA pellet was dried until color changed to blue after centrifugation at 14000 rpm for 45 min (4 °C). RNA was redissolved in 15 to 20 μl of pre-warmed RNAsecure (Ambion, USA). The samples were mixed and heated for 10 min (60 °C) to inactivate remained RNase. The concentration of nucleic acid was measured using a spectrophotometer (Synergy H1, BioTek Instrument Inc., USA), and the sample were diluted with RNAsecure. The synthesis of cDNA was performed using qScript cDNA synthesis kit (95047-100, Quantabio, USA) and then the samples were treated by PerfeCTa DNase kit (95150-01K, Quantabio, USA). IL-6 and IL-8 was measured using a real-time PCR instrument (CFX Connect optical reaction module, Bio-rad, USA).

**References**

1. Odum, J. R. et al. Gas/particle partitioning and secondary organic aerosol yields., *Envrion. Sci. Technol*. **30**, 2580-2585 (1996).
2. Borenfreund, E. & Puerner, J. A. A simple quantitative procedure using monolayer cultures for cytotoxicity assays (HTD/NR-90). *J. Tissue Cult Methods* **9**, 7-9 (1985).
3. Singh, N. P., McCoy, M. T., Tice, R. R. & Schneider, E. L. A simple technique for quantitation of low levels of DNA damage in individual cells. *Exp Cell Res*. **175**, 184-191 (1988).
4. Ames, B. N., McCann, J. &Yamasaki, E. Methods for detecting carcinogens and mutagens with the salmonella/mammalian-microsome mutagenicity test. *Mutat Res-Environ Mutag Related Subj* **31**, 347-363 (1975).
5. Organization for Economic Co-operation and Development (OECD). *Guideline for the Testing of Chemicals: (Part 471)* (1998).
6. Nadja, S. et al. A novel statistical approach for the evaluation of comet assay data. *Mutat Res Genet Toxicol Environ Mutagen* **652**, 1, 38-45 (2008).
7. Jiang, H., Jang, M., Sabo-Attwood, T., Robinson, S. E. Oxidative potential of secondary organic aerosols produced from photooxidation of different hydrocarbons using outdoor chamber under ambient sunlight. *Atmos Environ* **131**, 382-389 (2016).
